# Auto detection and segmentation of physical activities during a Timed-Up-and-Go (TUG) task in healthy older adults using multiple inertial sensors

**DOI:** 10.1186/s12984-015-0026-4

**Published:** 2015-04-11

**Authors:** Hung P Nguyen, Fouaz Ayachi, Catherine Lavigne–Pelletier, Margaux Blamoutier, Fariborz Rahimi, Patrick Boissy, Mandar Jog, Christian Duval

**Affiliations:** Département de Kinanthropologie, Université du Québec à Montréal, C.P. 8888, succursale Centre-Ville, Montréal, H3C 3P8 Québec Canada; Faculté des Sciences, Université du Québec à Montréal, Montreal, Canada; Electrical Engineering Department, University of Bonab, Bonab, East Azerbaijan Iran; Department of Surgery, Orthopaedic Division, Faculty of Medicine and Health Sciences, Université of Sherbrooke, Quebec, Canada; London Health Sciences Center University Hospital, Ontario, Canada

**Keywords:** Walk, Turn, Auto, Elderly, Activities of daily living, Optimization, Sit, Stand, Segment

## Abstract

**Background:**

Recently, much attention has been given to the use of inertial sensors for remote monitoring of individuals with limited mobility. However, the focus has been mostly on the detection of symptoms, not specific activities. The objective of the present study was to develop an automated recognition and segmentation algorithm based on inertial sensor data to identify common gross motor patterns during activity of daily living.

**Method:**

A modified Time-Up-And-Go (TUG) task was used since it is comprised of four common daily living activities; *Standing*, *Walking*, *Turning*, and *Sitting*, all performed in a continuous fashion resulting in six different segments during the task. Sixteen healthy older adults performed two trials of a 5 and 10 meter TUG task. They were outfitted with 17 inertial motion sensors covering each body segment. Data from the 10 meter TUG were used to identify pertinent sensors on the trunk, head, hip, knee, and thigh that provided suitable data for detecting and segmenting activities associated with the TUG. Raw data from sensors were detrended to remove sensor drift, normalized, and band pass filtered with optimal frequencies to reveal kinematic peaks that corresponded to different activities. Segmentation was accomplished by identifying the time stamps of the first minimum or maximum to the right and the left of these peaks. Segmentation time stamps were compared to results from two examiners visually segmenting the activities of the TUG.

**Results:**

We were able to detect these activities in a TUG with 100% sensitivity and specificity (n = 192) during the 10 meter TUG. The rate of success was subsequently confirmed in the 5 meter TUG (n = 192) without altering the parameters of the algorithm. When applying the segmentation algorithms to the 10 meter TUG, we were able to parse 100% of the transition points (n = 224) between different segments that were as reliable and less variable than visual segmentation performed by two independent examiners.

**Conclusions:**

The present study lays the foundation for the development of a comprehensive algorithm to detect and segment naturalistic activities using inertial sensors, in hope of evaluating automatically motor performance within the detected tasks.

## Background

With an increasingly aging population of older adults, promoting and maintaining a healthy mental and physical lifestyle is crucial for their quality of life. People suffering from motor degenerative diseases often experience limited mobility, which could lead to physical and mental deterioration further compounding the effects of aging [1,2]. Loss of mobility will manifest itself in activities of daily living (ADLs) through altered gait and increased the risk of falling [3]. Since, these limitations are felt during life activities, there is a need for a more systematic method of monitoring and evaluating the loss of mobility to increase the quality of life for older adults and people suffering from motor degenerative diseases.

Recently, inertial sensors have been used to detect human physical activities such as walking [4,5], lying [6,7] and falling in the elderly population [8], as well as in people with Parkinson’s disease [9-11]. The emphasis has been on the detection of activity to evaluate mobility both in clinical setting as well as in the home [6]. Sensor such as accelerometer has been widely adopted to detect physical activities due to its availability, compact size and low power consumption [4]. These sensors have been used to detect walking, sitting, and standing during the course of daily living [7,12], allowing measurement of performance parameters such as gait stride speed, stride length, etc. A system of inertial and barometric sensor on different anatomical locations has also been used to detect activities such as drinking and writing [12]. In addition to activity detection, postural transitions especially during *sit-to-stand* and *stand-to-sit* have been detected with high accuracy using a single chest mounted gyroscope [13] and tri-axial accelerometer [4]. However, the scope of these postural transition detections has been limited to static transition and the range of the activity that can be detected is limited by the amount of sensory information available.

These sensors have the potential to provide continuous mobility monitoring in the home environment, and therefore are more practical to deploy than laboratory based optical motion capture systems. The ultimate goal is to provide information that could be used to identify performance parameters to monitor disease or rehabilitation progress [14-16]. However, in order to remotely monitor performance, one must be able to segment, i.e., identify the subsets of movement within an individual task. Auto segmenting or isolating activities could then provide time stamps within which mobility parameters can be analyzed.

The objective of the present study was to develop and test an automated recognition and segmentation algorithm based on inertial sensor data to identify gross motor activities pattern in daily living tasks during a continuous trial. We used a modified Time-Up-And-Go (TUG) task as a model of simple activities that included four common activities; *Standing*, *Walking*, *Turning*, and *Sitting* performed in a continuous fashion.

## Methods

### Participants

Sixteen healthy, community dwelling older adults (9 females; 68.7 ± 9.3 years old, height =1.6 ± 0.1 m, weight = 62.8 ± 8.4 kg, BMI = 25.4 ± 3.5 kg/m^2^; 7 males, 67.3 ± 5.8 years old, height =1.7 ± 0.1, weight = 67.8 ± 9.5 kg, BMI = 23.4 ± 3.1 kg/m^2^) were recruited through the Centre de Recherche de l’Institut Universitaire de Gériatrie de Montreal (CRIUGM). Participants were screened for comorbidities and cognitive deficits. None of the participants exhibited any physical limitations or pain that could affect their ability to perform the task. The institutional research ethics review board of the CRIUGM approved this research and each participant read and signed an informed consent form.

### Experiment protocol

**Figure 1:**
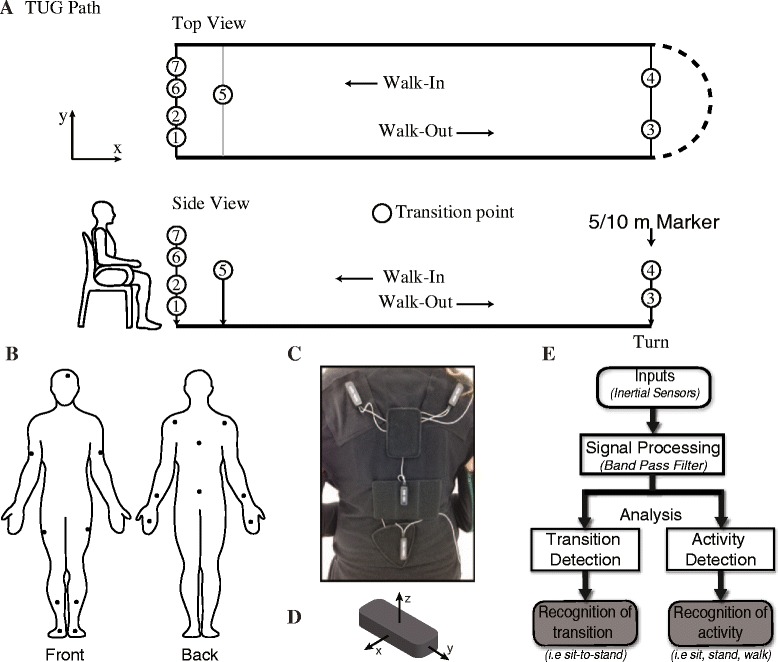
Schematic of the TUG task and the inertial sensor motion capture system. **A)** Spatial schematic of a TUG path and different transition points. Seven transitions were identified among the activities performed during a TUG. These transitions are: 1) *sit-to-stand* 2) *stand-to-walk-out* 3) *walk-out-to-turn* 4) *turn-to-walk-in* 5) *walk-in-to-turn* 6) *turn-to-sit* 7) *stand-to-sit*. **B)** Diagram of the 17 sensors and their location on the Animazoo suit. **C)** A close-up view of the sensors on the shoulders, trunk and hip. **D)** The orientation of the axes on the sensor. Using the right-hand Cartesian coordinate system, the y-axis line is aligned along the length of the inertial sensor while the x-axis is aligned along the width of the sensor. **E)** Global work flow of the algorithm to detect the activities and transition between activities using an inertial sensor motion capture system.

In this study, participants performed two randomly selected TUG tasks, one having length of 10 meters, the other 5 meters. Participants performed two trials of each TUG task. The algorithm was based on the 10 meters TUG because it provided more walking strides as well as a more gradual transition between *Walking* and *Turning*. The 5 meters TUG was used to evaluate the extensibility of the algorithm for shorter distance TUG task. The TUG was used simply because it contains key activities (*Standing*, *Walking*, *Turning* and *Sitting*) that are performed in a continuous fashion. Data recording started with participants in a standing position to align the sensors with the motion capture system, then sat down in a plastic armed-chair to perform the TUG task. Participants then stood up from the sitting position with their arms on the chair, walked to a distance marker on the floor, turned around, and walked back to the chair turned around, and finally sat down (Figure 1A). Participants were asked to perform these tasks at their own pace and no instructions were given on how to stand, sit, walk, or turn.

Participants performed these TUG tests while wearing the Animazoo IGS-180 motion capture suit (Synertial UK Ltd, Brighton, UK). The ISG-180 (Figure 1B, C) is equipped with 17 inertial sensing modules (OS3D, Inertial Lab, VA, USA) positioned on each limb in order to capture full-body 3D movement. Each sensor module is comprised of 3-axis linear acceleration (accelerometer), angular velocity (gyroscope) and magnetic north heading (magnetometer). Raw data (acceleration, angular velocity) and fused data (3D orientation in degrees estimated from a fusion algorithm [17-20] developed by Animazoo) from each sensor were acquired at 60 Hz. Since there was no *a priori* expectation as to which sensors were suitable markers for detection and segmentation, all 17 inertial sensors were active during the recording.

### Sensor location

The head sensor was attached to a cap worn by the participants, which positioned it on the right side of the head. The trunk sensor was located on the midline over T1, while the hip sensor was positioned at the level of L5. For upper extremities, shoulder sensors were positioned over the scapula; upper arm sensors were positioned between the shoulder and elbow while forearm sensors were positioned between the elbow and wrist joint. Hand sensors were attached to an open-finger glove, and positioned on the dorsum surface of the hands. In the lower extremity, thigh sensors were positioned on the outer side of the limb segment between the hip and knee. Shin sensors were positioned between the knee and ankle. Foot sensors were attached to the dorsum of the shoes worn by the participants.

### Signal conditioning

The signals from the inertial sensors were detrended to remove sensor drift and normalized against the absolute maximum amplitude of each signal (unitless) to ensure uniformity in the analysis across all participants. Ideal band pass filters in the frequency domain were applied. An ideal frequency-selective filter is a system that passes a pre-specified range of frequency components without any attenuation, but completely rejects the remaining frequency components. The transfer function of the ideal pass band filter is defined as follows:

1 $$ {H}_{BP}(jw)=\left\{\begin{array}{cc}\hfill 1\hfill & \hfill {w}_1\le w\le {w}_2\hfill \\ {}\hfill 0\hfill & \hfill elsewhere\hfill \end{array}\right. $$

Where *w*_*1*_ and *w*_*2*_ are referred to as the low and high cutoff frequencies, respectively. A band pass filter was chosen and constructed as a generalize filter for the different sensors in the motion capture system. The band pass filter has a finite bandwidth as it only allows a range of frequencies (*w*_*1*_ ≤ *w* ≤ *w*_*2*_) to be passed through the filter. The dominant frequencies in these inertial sensors during a TUG (sampled at 60 Hz) were less than 10 Hz. The low cut frequency was set at *w*_*1*_ = 0.0025 Hz to capture all the low frequency dynamics and to condition the data in the frequency domain by removing the fundamental frequency and centralizing the data. The high cut frequency was optimized for each sensor (*w*_*2*_ < 10 Hz) with an exhaustive search optimization method using the time stamps from the inertial sensors and the visual segmentation (see below). However, the cutoff frequency of the hip angular velocity used for the detection of *Walking* was set at the Nyquist frequency (30 Hz) to capture the stride information during walking. At low cutoff frequency, the stride features during *Walking* would not be detectable.

### Sensor selection

#### Activity detection

**Figure 2:**
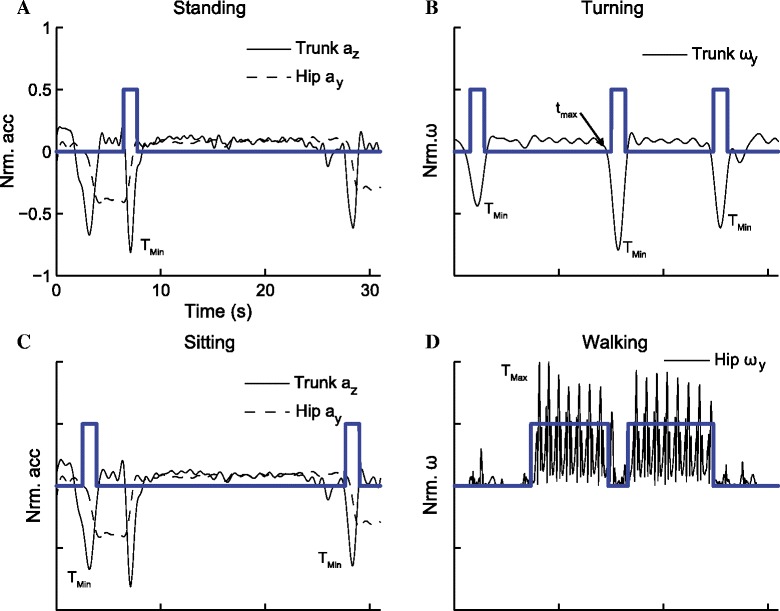
Activities detection during a TUG. Detection of different activities during a TUG for one participant. Raw signals were detrended and normalized for uniformity across all participants. The detection algorithm relied on detecting the large kinematic peaks in the inertial sensor (T_Max_ and T_Min_) that corresponded to different activities. The square signals were generated at 30% of the peaks to visually indicate that activities were detected during TUG. **A)** *Standing,* when participants stand up from the chair, was detected using the trunk a_z_ and the time derivative of the hip (${\dot{a}}_y\kern0.5em >0$). **B)** *Turning* was identified using the trunk angular velocity (ω_y_) **C)** *Sitting*, when participants sit down on the chair, was detected using the trunk a_z_ and the time derivative of the hip (${\dot{a}}_y\kern0.5em >0$). **D)** *Walking* was identified by using a 500 ms windows to detect the oscillation in the angular velocity of the hip (ω_y_).

**Figure 3:**
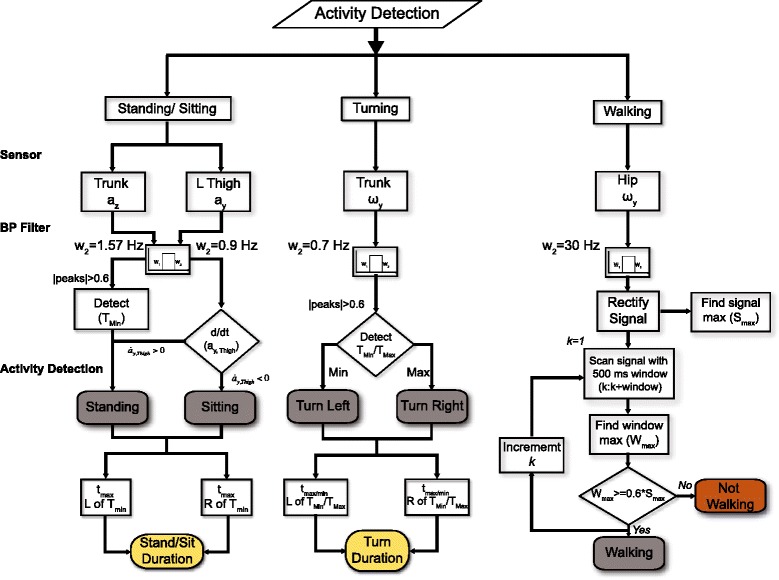
Flow chart of the detection algorithm use to identify the scripted activities during a TUG. These sensors were normalized and detrended for uniformity across all participants. The high cut frequencies of the band pass filter were determined by optimizing the difference between the transition time using the inertial sensors and by visual inspection. T_Max_ or T_Min_ denotes the large peaks that correspond to different activities while t_min_ or t_max_ represents the first minimum or maximum to the left or right of T_Max_ or T_Min_.

The sensors selected for activity detection were based on how they corresponded to the biomechanics of movement during the performance of these activities. *Standing* which denotes when participants stand up from the chair was detected using the acceleration of the trunk (a_z, Trunk_). *Sitting* which denotes when participants sit down on the chair was also detected using the same sensor data. Sensors on the trunk or chest have been used to identify *Standing* and *Sitting* during physical activities [4]. However, in this study, the time derivative of the acceleration $\left({\dot{a}}_{y, Hip}\right)$ of the thigh was also used to differentiate between *Standing* and *Sitting*. During *Standing*, ${\dot{a}}_{y, Hip}\kern0.5em >0$ and during *Sitting*, ${\dot{a}}_{y, Hip}\kern0.5em  < 0$. The angular velocity (ω_y, Trunk_) of the trunk was used to detect *Turning*. The angular velocity of the head (ω_y, Head_) was also used to verify that *Turning* has occurred and the direction of *Turning. Walking* was detected by using a 500-millisecond window to detect the oscillation in the angular velocity (ω_x, Hip_) of the hip. *Walking* was also detected during *Turning*; however priority was given to classify this as *Turning*. The detections of *Standing*, *Turning*, *Sitting*, and *Walking* are shown in Figure 2. The activities were detected by finding the maximal or minimal peaks of the selected sensors that corresponded to different activities. The square signals were generated by setting the threshold at 30% of peak amplitude to provide visual indication that an event was detected. The algorithm and sensors used to detect the activities during a TUG are shown in Figure 3.

#### Segmentation

Four common daily living activities were featured during a TUG task. These activities are: *Standing*, *Walking*, *Turning*, and *Sitting*. The sequence of these activities generated six different *segments* during a TUG task. These segments were: *Stand up*, *Walk-out*, *Turn 180*, *Walk-in*, *Turn 180* and *Sit down*. The transition point was defined as the separation point between two consecutive segments. The transition points between these segments were identified by detecting the time stamp of the first minimum or maximum to the left and right of the segment peak, which marked the beginning and ending of each segment in the TUG. The seven transitions identified during a TUG are: *sit-to-stand*, *stand-to-walk-out*, *walk-out-to-turn*, *turn-to-walk-in*, w*alk-in-to-turn*, *turn-to-stand* and *stand-to-sit*.

**Figure 4:**
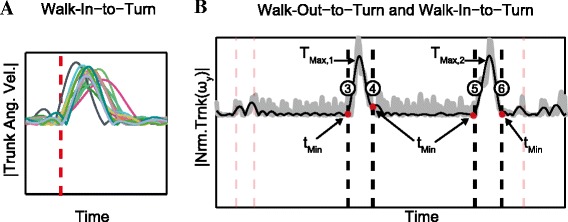
Temporal schematic of segment transitions during a TUG and kinematics pattern during turning transitions. **A)** Selected inertial sensors are identified on the Y-axis (trunk ω_y_) of the graphs and the kinematics pattern during a *walk-in-to-turn* transition for all participants (n = 16). These patterns showed a consistent kinematic behavior of this sensor during *Turning*; therefore, it was used to identify *Turning* as well as the transition to the activities before and after *Turning*. **B)** The raw and filtered signals of the trunk ω_y_ with two different maximum peaks that indicated two different turns during the TUG task. The time stamps of first minimum peak to the left and right of these peaks were used to approximate the transition point before and after *Turning*.

**Figure 5:**
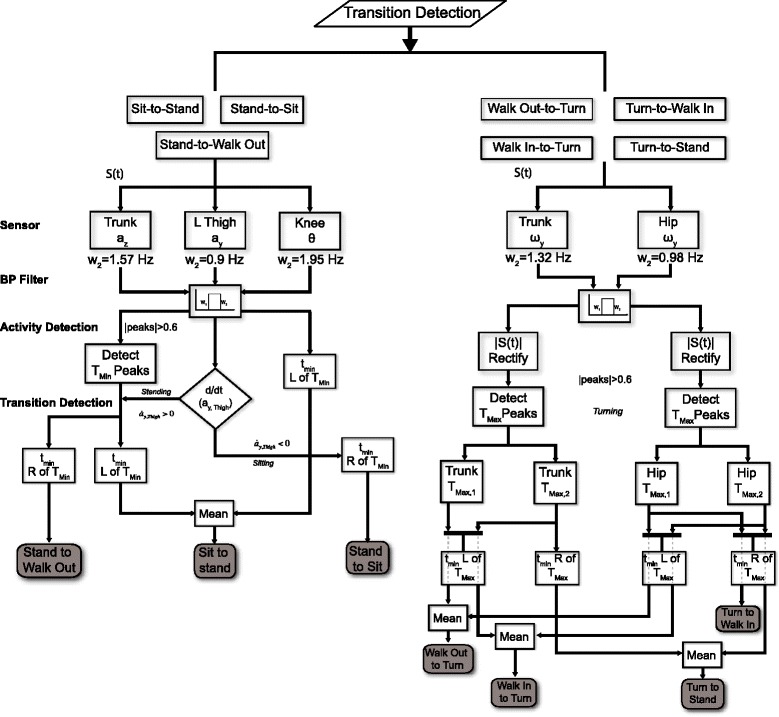
The sensors and algorithm used to segment different transition points during a TUG. These sensors were selected by optimizing the transition time from each sensor with the visually segmented time. *Sit-to-stand* transition was detected using mean time from the acceleration of the trunk (a_z_) and the knee angle (*θ*) while *stand-to-sit* and *stand-to-walk-out* were estimated using only the trunk a_z_. Transitions before and after *Turning* were detected using the mean time estimated using the ω_y_ of the hip and the trunk. However during *turn-to-walk-in* transition only the ω_y_ of the hip was used since it yielded the best approximation as compared to the visual segmentation.

The kinematic pattern of the joints and limbs during the performance of these activities were used to identify a set of sensors that marked the transition point for each segment. For example, the patterns of the trunk angular velocity for all participants during *walk-in-to-turn* are shown in Figure 4A. While there were variability between participants in the duration and amplitude of these signals, there was a similar pattern that indicated the beginning and ending of *Turning.* While the maximal peak in trunk angular velocity (ω_y, Trunk_) was used to detect *Turning*, the time stamp of the first minimum to the left and right (t_min_) of these peaks were used to approximate the transition between *Walking* and *Turning* (Figure 4B). Similar patterns were also exhibited in the hip and head sensor. However, these sensors were not always in-phase with each other; therefore, some might have lagged while others led. Therefore, an average of the sensor information from the head, trunk and hip were use as surrogate approximation of the *walk-to-turn* and *turn-to-walk* transition. The transition time for a few selected sensors were individually and collectively (using the mean) compared with the visual segmentation time and the sensor combinations that yielded the smallest differences across all participants were used to estimate the transition between these activities. The selected sensors and the algorithm to detect these transitions are presented in Figure 5.

### Visual segmentation

**Figure 6:**
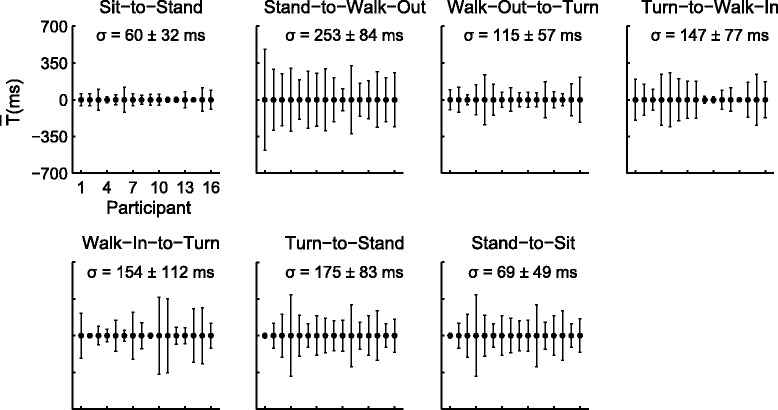
Variance of the visual segmentation of different transition points during a 10 m TUG between two examiners. The variance (mean ± std) from the visual segmentation of the different activities in a TUG for all participants (n = 16) at all transition by two independent examiners. The segmentation was marked by using the avatars provided by Animazoo. Participant performed two trials of the 10 meter TUG.

In addition to the development of an algorithm for the automatic detection and segmentation of activities performed during a TUG, we were also interested in determining if the transition points detected by the segmentation was at least as accurate as visual segmentation done by simply looking at the avatar. To do so, two examiners were asked to independently segment these activities during the 10 meter TUG using the avatar generated by the software during the performance of the task to provide validation to the automated algorithm. These two examiners were provided with general transition segmentation guidelines and were instructed to mark the time stamp of when the participants began to transition into different activities during the TUG. The variability of the marked time between two examiners for all participants (n = 16) and all transitions (n = 224) is shown in Figure 6. The variability between the two examiners was then used to evaluate the performance of the algorithm in estimating the transition points using the information from the sensors for all participants. The examiners were most variable when marking the transition time during *stand-to-walk-out* while they were less variable during *sit-to-stand* transition. The *sit-to-stand* was easier to identify because it started from a static sitting position while other transitions were dynamically blended from one movement to the next. This was also evident during *stand-to-sit* transition. During dynamic transition between different activities within a TUG, it was more difficult for two examiners to agree on exactly when the activities started and ended because of how differently participants performed the TUG. Furthermore, these variations between the examiners were also evident in individual participant. These examiners were more variable in some participants than others during segmentation of the same transition. This indicates that participants did not perform the task in exactly the same way, which might affect the judgment of the examiners as to when the transition started and ended. The differences in how participants performed the task might contribute to the variability between the examiners. Since participants were performing the TUG at their own pace, the time stamp $\left(\overline{T}\right)$ was shifted to zero to normalize the marked time across all participants. It took approximate 7 minutes to visually segment one 30-second trial.

### Range of Motion (ROM) calculation

In addition to using the velocity and acceleration profiles of each sensor for the automatic detection and segmentation of activities performed during a TUG, we also considered the orientation data originating from the fusion algorithm of the sensors. To do so, quaternions were used to calculate the angles between limb segments, for instance, the angle between the hip and the thigh (hip ROM) or the angle between the thigh and the tibia (knee ROM). To calculate these angles, we used the quaternion output, which is a four-dimensional scalar and vector representation of complex numbers,

2 $$ q=\left[w\ x\ y\ z\right] $$

Where *w* is a real number and v = [*x y z*] is a vector

For example, let *q*_1_ and *q*_2_ represent the quaternions of the hip and thigh respectively and *q*_rel_ defines the relative quaternion between these two segments, then

3 $$ {q}_{rel}={q}_1^{-1}*{q}_2 $$

To track the relative changes of a quaternion during the TUG, a reference quaternion, *q*_*ref*_, was recorded at the start of each trial when participants were in a standard pose position with their arms along the sides. The change in the quaternion was defined as

4 $$ {q}_{\Delta}={q}_{ref}^{-1}*{q}_{rel} $$

Post-processing algorithms were applied to *q*_∆_ to ensure small angle representation (less than 180°) and continuity in the signal. The range of motion of the hip and knee were calculated by taking the real part of the inverse cosine of the quaternions.

5 $$ ROM= real\left(2{ \cos}^{-1}\left({q}_{\Delta}\right)\right) $$

### High cut frequency optimization

The high cut frequency for the band pass filter (*w*_*2*_) was found my minimizing the sum of the square difference between the transition time stamp acquired using the inertial sensor and visually by two examiners across all participants (n = 16).

6 $$ \mathrm{minimize}\left\{{\displaystyle \sum_{k=1}^{16}{\left({T}_{manual,k}-{T}_{Sensor,k}\right)}^2}\right. $$

**Figure 7:**
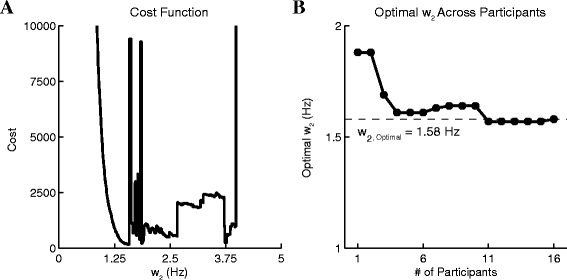
Optimization of *w*_*2*_ of trunk a_z_ during *sit-to-stand* transition. **A)** The cost function of the trunk a_z_ as a function of the high cut frequency (*w*_*2*_) during *sit-to-stand* transition across all participants (n = 16). **B)** The convergence of *w*_*2*_ as it optimized across more participants. This result indicated that kinematics patterns were stable in these inertial sensors during the performance of these activities. While there was variability between participants, the optimal frequency quickly converged when more participants were factored into the cost function. Similar behaviors were also observed in other sensors for all seven transitions.

The objective function of the optimization problem to find the high cut off frequency is shown in eq. 6, where T_visual, *k*_ was the mean time estimated by both examiners and T_sensor,*k*_ was the time estimated by the inertial sensors(s) and *k* was an index for the participants. This was done for all selected sensors for all seven transitions. The sensor or combinations of sensors (mean) that yield the lowest cost function across all participants were selected to approximate the transition time. An example of the cost function of the trunk acceleration (a_z_) as a function of the high cut-off frequency is shown in Figure 7A.

An exhaustive search optimization method [21] was used to find the high cutoff frequency for the inertial sensor (0.5 Hz ≤ *w*_*2*_ ≤10 Hz). This frequency band corresponded to the dominant frequencies of the activities performed during a TUG. 2000 steps between this frequency band were used to find the optimal high cut frequency. The optimal high cutoff frequencies for each sensor during each transition are summarized in Table 1.

**Table 1:** **Optimal high cutoff frequency for each sensor at different transitions during a TUG task**

**Transition**		**Sensor**					
		**1**	**Freq (Hz)**	**2**	**Freq (Hz)**	**3**	**Freq (Hz)**
1.	Sit-to-Stand	Trunk a_z_	1.57	Hip ω_x_	0.69		
2.	Stand-to-Walk	Trunk a_z_	2.44	L Knee ROM	8.30		
3.	Walk-to-Turn	Trunk v_y_	1.32	Head ω_x_	0.79	Hip v_y_	0.98
4.	Turn-to-Walk	Hip v_y_	0.53	Head ω_x_	0.41		
5.	Walk-to-Turn	Trunk v_y_	1.00	Hip ω_y_	0.59		
6.	Turn-to-Stand	Trunk v_y_	1.00	Hip ω_y_	0.81		
7.	Stand-to-Sit	Hip a_z_	1.07				

### Independent measures

Sensitivity and specificity [22] were used to evaluate the performance of the algorithm to auto detect the activities performed during a TUG. Sensitivity measures the proportion of actual positive activities detected (true positive) while specificity measures the proportion of the negative activities that were detected (true negative).

The means of the absolute differences (ΔT) and the variances (σ) between the transition time segmented visually by two examiners and automatically using the inertial sensors were used to evaluate the performance of the algorithm across sixteen participants at each transition. The time difference at each transition was defined

7 $$ \Delta T=\frac{1}{16}{\displaystyle \sum_{k=1}^{16}\left|{T}_{visual,k}-{T}_{Sesnor,k}\right|} $$

Where *k* was an index for the numbers of participant.

## Results

The aims of this work were to develop an algorithm to utilize data from inertial sensor to detect activities such as *Standing*, *Sitting*, *Walking* and *Turning* as well as isolating these activities for post analysis of performance.

### Segment detection

The data analysis on 16 participants performing two trials of a 5 and 10 meter TUG task yielded 384 (16 participants × 6 segments × 2 trials × 2 tasks) instances of activities such as *Standing*, *Sitting*, *Walking*, and *Turning*. Using the algorithm with the selected sensors, the proposed algorithms were able to detect the activities with 100% sensitivity and specificity during the 10 meter TUG (n = 192). To validate the generality of the algorithms in detecting these activities, we then proceeded to test the *same* algorithms and parameters on the data recorded during the 5 meter TUG (n = 192). Participants performing the 5 meter TUG displayed similar kinematic patterns with half the duration (~15 vs. ~28 seconds). Again, without changing any of the parameters, the algorithm was also able to detect these activities with 100% sensitivity and specificity.

### Transition detection

**Figure 8:**
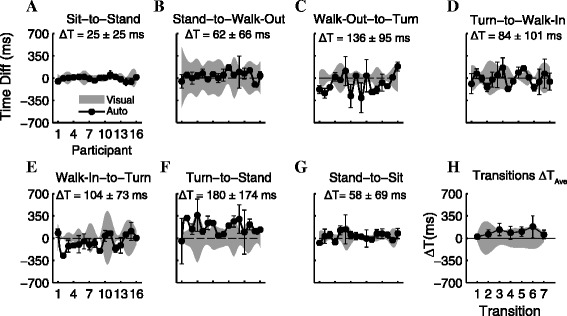
Differences between visual and auto segmentation of transition time during a 10 m TUG. **A-G)** The time stamp differences (mean ± std) of individual participant and of each transition (ΔT). This was the difference between the transition time marked visually by two examiners and the time detected using the inertial sensors. **H)** On average (n = 16), the absolute difference between the times marked visually and using inertial sensors were within one standard deviation of each other for all transitions.

When applying the segmentation algorithms to the 10 meter TUG, we were able to parse 100% of the transition points (n = 224) between different segments. The differences and variances between the visual and auto segmentation of a 10 meter TUG across all transition points for all participants (n = 16) are shown in Figure 8A-G. The smallest variability across all participants using inertial sensors was during the *sit-to-stand* transition (σ = 25 ms, Figure 8A) while the largest variability was during the *turn-to-stand* transition (σ = 174 ms, Figure 8F). In comparison, the smallest variability during visual segmentation between the two examiners was also during *sit-to-stand*; however, the variance was larger (σ = 60 ms). During *stand-to-walk-out* transition, the estimated transition time was more variable when marked visually by two examiners (σ = 253 ms) than using the inertial sensors (σ = 66 ms, Figure 8B). On average, the automated segmentation $\left(\overline{\sigma}{}_{Auto}\kern0.5em =\kern0.5em 86\kern0.5em  ms\right)$ was less variable than the visual segmentation $\left(\overline{\sigma}{}_{Auto}\kern0.5em =\kern0.5em 86\kern0.5em  ms\right)$ across all participants and transitions.

The smallest difference between the visual and auto segmentation was during the *sit-to-stand* transition (ΔT = 25 ms) while the largest was during the *turn-to-stand* transition (ΔT = 180 ms). Across 7 transitions, the average difference between the visual and auto segmentation was approximately ΔT_ave_ = 93 ms. The estimated transition time across all participants approximated using the inertial sensors were within one standard deviation of the transition time marked visually by two examiners.

## Discussion

The aims of this work were to develop an algorithm that utilized the information from an inertial sensor-based motion capture system to identify gross physical activities performed during daily living and automatically isolate these activities for future performance analysis. This was accomplished by using an optimization method to filter and identify body-worn sensors in the system that were strongly associated to different activities and yielded the best performance. The results from the optimization lead to the development of an efficient detection and segmentation algorithm that minimized the effect of movement variability between participants while robustly detect and segment the activities during a TUG. This study has also demonstrated that using a set of inertial sensors and applying the detection algorithm, it was possible to identify and segment these activities during continuous execution of daily activity tasks in a healthy older adult population with 100% accuracy.

Attempting to detect specific activities is not a new concept. For instance, Torres *et al.* [12] using a module of inertial and barometric sensors place at different location of the body were able to detect walking at 100% and sit and stand at 86% and 89%, respectively. Godfrey *et al.* [4] used a chest mounted accelerometer to detect *Standing* with sensitivity and specificity of 83% (±11) (mean ± SD, n = 10)). While Najafi *et al.* [13] could detect the same activity with more than 95% sensitivity and specificity in healthy elderly using a miniature gyroscope during long-time monitoring. In the present study, using a combination of pertinent information from specific sensors, we were able detect these activities with 100% specificity and sensitivity.

Since participants were told to perform the task at their own pace, there was variability in how they performed the TUG task. In fact, an older population was specifically selected because of its inherent variability in performing tasks, in addition to be the type of population that is more often the subject of mobility assessment. Using the optimal approach to find the cutoff frequencies, we minimized the effect of variability between participants by generating a single set of parameters (cutoff frequency) that can be applied to all participants. The global convergence of these cutoff frequencies indicated that kinematic patterns generated by the participants during the performance of the TUG were very similar (Figure 7B).

Segmentation of these activities during daily living will become crucial when this type of sensors are deployed remotely in homes and free environments for long-term monitoring of patient’s mobility. Also, segmentation using the avatar is hugely time consuming. Case in point, we have asked an examiner to segment a five-minute free-living mobility activity of a person moving in an environment where they were able to perform multiple tasks. It took the examiner 5 hours to visually identify the different segments and time-stamp the transition points. While general guidelines were presented to the examiners on how to segment this task, there was still a large variability between the examiners on determining the onset and end of segments, especially during dynamic transitions (Figure 6). In general, the transition points detected by the algorithm were less variable than visual segmentation across all transition points and participants (Figure 8). If we are to assume that times marked by visual inspection is the gold standard*,* then largest time difference between the visual and automatic segmentation were approximately 180 *ms* during the most challenging transition, *turn-to-stand*. Given that the average variance between the examiners were 175 *ms* during this transition, the difference between visual and automatic segmentation would not be significant; yet, the algorithm was significantly faster in detecting these activities and segmenting the TUG task.

Detecting if a person stands up or sits down is critical for monitoring and evaluating *how* well the person has performed that task. For example, the time it takes for patients to perform a *sit-to-stand* task has been correlated with the risk of falling as well as functional recovery in community dwelling elderly [15,16]. Cheng [23] showed that time needed to complete a 180° turning was a good index to differentiate between fallers and non-fallers in individual with Parkinson’s disease (PD). Stack [24] showed that, on average, people with PD took more steps during turning to compensate for the difficulties experienced during turning. The present study provides an automated method to quickly isolate out these activities using inertial sensors. Such segmentation will be used in the future to assess the quality of the mobility for the detected tasks.

## Conclusion

The present study lays the foundation for the development of a comprehensive algorithm to detect and segment activities performed during daily living using inertial sensors. The current study is limited in scope by the relatively simple tasks that were segmented, the environment in which the tasks were performed, and the relatively healthy population that performed the tasks. We are currently applying the detection and segmentation principles to less scripted tasks and in more unstructured environments, with longer trial durations. We are also testing our algorithms on populations with altered mobility. We expect that introducing more complex tasks and in a more variable environment and population would probably require more sensors (redundancy) to detect and segment the tasks. This is why we always record the tasks with 17 sensors, in the hope of providing the optimal sensor set for specific conditions. We also suspect that further optimization will be required when populations with altered mobility are studied. Nonetheless, the current results lay the foundation for future research, and could be utilized to develop a fully-automated TUG capture and analysis system. Ultimately, the detection and segmentation of these activities is needed to develop performance metrics to evaluate and monitor people with mobility impairment due to disease and old age.

## Abbreviations

TUG: Timed-up-and-go test
ADL: Activities of daily living
